# Defining the Ideal Patient with Hepatocellular Carcinoma for Second-Line Treatment

**DOI:** 10.1155/2020/8024124

**Published:** 2020-06-19

**Authors:** Giandomenico Roviello, Andrea Casadei-Gardini, Stefania Nobili, Enrico Mini, Sara Fancelli

**Affiliations:** ^1^Department of Health Sciences, University of Florence, Viale Pieraccini, 6, 50139 Florence, Italy; ^2^Division of Oncology, Department of Medical and Surgical Sciences for Children & Adults, University-Hospital of Modena and Reggio Emilia, 41121 Modena, Italy; ^3^School of Human Health Sciences, University of Florence, Largo Brambilla 3, 50134 Florence, Italy

## Abstract

**Background:**

Second line of treatment of hepatocellular carcinoma (HCC) has notably changed in recent years as three novel drugs with a different mechanism of action have demonstrated to improve survival compared to placebo; thus, there is a need to better define the profile of optimal candidates to second-line treatment with these drugs in order to maximize clinical benefit.

**Materials and Methods:**

We performed a pooled analysis from the subgroup analysis of all published phase III trials for approved targeted therapy in the second line of treatment for HCC, with the aim to discover possible clinical-pathological predictive factors.

**Results:**

Four studies were included in the analysis for a total of 2137 cases whose results supported the use of these novel agents in male patients with ECOG: 0, extrahepatic metastases, and HBV infection.

**Conclusions:**

Future studies are awaited to define best candidates for novel agents approved in the second line of treatment for HCC.

## 1. Introduction

Hepatocellular carcinoma (HCC) is the sixth most commonly diagnosed cancer worldwide and one of the main causes of cancer-related deaths worldwide [1]. Generally, patients with early stage of disease are optimal candidates to potentially curative treatment such as surgery, transplantation, and ablation [2]. Additionally, chemoembolization should be taken into consideration for patients with normal liver function and localized disease [2]. For patients progressed after this treatment or no longer susceptible to locoregional therapy, the oral multikinase inhibitor sorafenib has been the first drug to improve overall survival (OS) over placebo and has been considered the standard of care for many years [3, 4]. In parallel, based on the positive results of the phase III noninferiority study REFLECT, lenvatinib, another oral multikinase inhibitor, has become an alternative option to sorafenib in first line of therapy [5].

Until recently, for patients who progressed to sorafenib, there has been no validated option. However, in the last years, this scenario is greatly changed, as novel drugs have demonstrated to improve survival compared to placebo in the second line of treatment [2]. These drugs included the monoclonal antibody ramucirumab and the multityrosine kinase inhibitors regorafenib and cabozantinib. Although all these drugs have enlarged the spectrum of available options for the management of the second line of treatment of advanced HCC, there is still the need to better define the profile of optimal candidates to these drugs in order to maximize clinical benefit. Therefore, we performed a pooled analysis from the subgroup analysis of all published phase III trials that reported positive results of novel drugs in the second line of treatment for HCC, with the aim to discover possible clinical-pathological predictive factors.

## 2. Methodology

The studies for this analysis were chosen between those approved targeted therapy of patients with HCC in the second line of treatment. In particular, the following inclusion criteria were identified: (1) participants with HCC treated in the second line of therapy; (2) possibility to assess HR for survival; and (3) possibility to assess subgroup analysis in the study trials. The following exclusion criteria were used: (1) insufficient availability of data estimating the outcomes; (2) the presence of a single-arm study; and (3) no data on subgroup analysis. Study quality was assessed using the Jadad 5-item scale, considering randomisation, double-blinding, and withdrawals. The final score ranged from 0 to 5 [6]. The summary estimates were generated using a fixed-effect model (Mantel–Haenszel method) or a random-effect model (DerSimonian–Laird method) [7, 8], depending on the absence or presence of heterogeneity. Statistical heterogeneity was assessed with the *Q*-test and the *I*^2^ statistic. *I*^2^ values of 25%, 50%, and 75% were considered to indicate low, moderate, and high heterogeneity, respectively [9]. When *P* > 0.1 and *I*^2^ < 50%, the fixed-effects model was used; otherwise, the random-effects model was used. A value of *P* < 0.05 was regarded as statistically significant, and all tests were two-sided.

## 3. Results

Four studies were included in the analysis [10–13] for a total of 2137 cases (1329 cases in the experimental group and 808 cases in the control group). The characteristics of the trials are reported in Table 1. For analysis of baseline clinical-pathological factors age, sex, tumour location, sex, ECOG performance status, alpha-fetoprotein values, macrovascular invasion, extrahepatic metastases, and viral infection status were considered; data on hazard ratio and confidence interval of overall survival (OS) for different subgroup analysis are reported in Table 1S.

The analysis of OS according to age showed that experimental drugs significantly improved OS in both patients aged ≥65 years and those aged <65 years (Table 2). When we stratified patients according to sex and ECOG, we found that OS was significantly higher in the experimental arm versus the control arm in male patients (HR = 0.76; 95% CI: 0.68–0.85; *P* < 0.001) compared to female (HR = 0.79; 95% CI: 0.60–1.05; *P* = 0.10) (Table 2) and that OS was significantly higher in the experimental arm versus the control arm in ECOG: 0 patients (HR = 0.70; 95% CI: 0.60–0.80; *P* < 0.001) compared to ECOG: 1 (HR = 0.88; 95% CI: 0.75–1.03; *P* = 0.11). Similar efficacy was demonstrated for the presence of extrahepatic metastases and HBV virus (Table 2). Finally, alpha-fetoprotein values (cutoff: 400 ng/mL) and macrovascular invasion (present or absent) were both predictors of survival in patients treated with experimental arms (Table 2). Supplementary files reported a pooled analysis of all clinical-pathological factors (supplementary files).

## 4. Conclusion

Treatment of patients with advanced HCC who progressed after a standard anti-antiangiogenetic first line of treatment is an evolving scenario with several open questions and doubts. In the REACH trial, the anti-VEGFR2 monoclonal antibody ramucirumab has shown a survival benefit in the prespecified subpopulation of patients with elevated baseline *α*-fetoprotein concentrations of 400 ng/mL or greater [10]. This was subsequently confirmed in the confirmatory REACH-2 trial that enrolled only patients with baseline *α*-fetoprotein concentration of 400 ng/mL or greater [13]. In addition, both regorafenib, a VEGFRs, PDGFRs, KIT, and Tie2 inhibitor, and cabozantinib, a VEGFRs, MET, and AXL inhibitor, have shown to increase survival compared to placebo in the RESOURCE and CELESTIAL trials, respectively [11, 12]. Pembrolizumab deserves a separate comment; indeed, despite the encouraging results of phase II clinical trial which accelerates its FDA approval in the second line, phase III KEYNOTE-240 has not reached the primary endpoint in OS [14, 15]. Unfortunately, data from the analysed studies showed that the absolute survival gain (difference between OS of the experimental arm with the OS of placebo) ranges from 1.2 to 4.2 months (Table 2). Based on these data, there is an urgent need to better identify and select patients who may benefit from therapy with cabozantinib, ramucirumab, and regorafenib as the second line of treatment in HCC. Among the most relevant prognostic factors, we found the ECOG performance status and the presence of macrovascular invasion (Table 2). Other absolute prognostic factors were sex in favour of male patients and the HBV infection (Table 2). In addition, the impact of age (with a cutoff of 65 years old) seems not so relevant in the prediction of OS and the value of alpha-fetoprotein ≥400 ng/ml correlates with the best clinical outcomes for all patients treated with novel agents and not only for patients treated with ramucirumab.  Table 3 reports possible preferential eligibility parameters for selecting HCC patients for second-line treatment with these novel agents.

Unfortunately, although the total sample size in our work is large, this analysis presents several limitations that we ought to consider: first of all, the analysis of data was literature-based rather than an analysis of raw data; in addition, the number of trials investigated (four) was rather limited and the heterogeneity of data was high, mainly due to the nature of the study trials with different patients' characteristics and with different experimental agents. The absence of an active comparator was also another relevant drawback. In addition, the studies for this analysis were chosen between those that reported positive outcomes for OS of patients with HCC in the second line of treatment: we recognize that this is of course a selection bias; thus, we cannot yield safe conclusions as instead a meta-analysis with a rigorous previous systemic review could have permitted. For all these reasons, definitive conclusions should be considered with caution. Moreover, recently, the phase III trial ImBrave150 demonstrates a statistically significant improvement in both OS and PFS in patients with unresectable HCC from the combination of atezolizumab plus bevacizumab in patient who never underwent prior line therapies. Those results have the potential to change in a near future the clinical practice in first-line setting locally advanced HCC patients [16].

However, in the absence of prospective data and with a lack of applicability of new combination in the first-line setting, our analysis involving more than 2000 cases seems to support the use of these novel agents in male patients with ECOG: 0, extrahepatic metastases, and HBV infection. Meanwhile, future studies in this setting are awaited to confirm this clinically address and to find biomolecular predictors of efficacy, especially for the angiogenesis and other druggable targets, useful in clinical practice.

## Figures and Tables

**Table 1:** Characteristics of the analysed trials.

Study	Design	Primary endpoint	Number of patients in the experimental arm	Number of patients in the control arm	Experimental drug (OS)	Control arm (OS)	Delta OS
CELESTIAL	III	OS	470	237	Cabozantinib (10.2 months)	Placebo (8.0 months)	2.2 months
REACH	III	OS	283	282	Ramucirumab+ BSC (9.2 months)	Placebo+ BSC (7.6 months)	1.6 months
REACH-2	III	OS	197	95	Ramucirumab+ BSC (8.5 months)	Placebo+ BSC (7.3 months)	1.2 months
RESOURCE	III	OS	379	194	Regorafenib+ BSC (10.6 months)	Placebo+ BSC (7.8 months)	2.8 months

OS: overall survival; BSC: best supportive care.

**Table 2:** Subgroup analysis of ramucirumab, regorafenib, and cabozantinib compared to placebo in HCC.

	HR	95% CI	*P* value	*I* ^2^ (%)	*P* value	Model

Age ≥65 years	**0.75**	0.64–0.88	<0.001	0	0.79	Fixed
Age <65 years	**0.80**	0.69–0.93	<0–001	9	0.35	
Male	**0.76**	0.68–0.85	<0.001	27	0.25	Fixed
Female	0.79	0.60–1.05	0.10	0	0.56	
ECOG: 0	**0.70**	0.60–0.80	<0.001	0	0.57	Fixed
ECOG: 1	0.88	0.75–1.03	0.11	0	0.11	
Alpha-fetoprotein						Fixed
<400 ng/ml	**0.85**	0.73–1.00	0.05	68	0.05	
≥400 ng/ml	**0.70**	0.69–0.85	<0.001	0	0.96	
Macrovascular invasion						Fixed
Yes	**0.75**	0.62–0.91	<0.001	0	0.66	
No	**0.75**	0.66–0.86	<0.001	20	0.29	
Extrahepatic metastases						Fixed
Yes	**0.70**	0.62–0.80	<0.001	0	0.44	
No	1.02	0.82–1.27	0.86	0	0.70	
Virus						Fixed
HBV	**0.71**	0.60–0.84	<0.001	0	0.52	
HCV	0.89	0.72–1.12	<0.32	0	0.67	

HR: hazard ratio; CI: confidence interval.

**Table 3:** Eligibility criteria for overall survival benefit with ramucirumab, cabozantinib, and regorafenib as the second line of treatment for HCC.

Main
Male patients
ECOG: 0
Extrahepatic metastases
HBV infection

Independent
Age
Alpha-fetoprotein
Macrovascular invasion

## Data Availability

The data used to support the findings of this study are available from the corresponding author upon request.

## Supplementary Materials

Supplementary Materials Figures of subgroup analysis for overall survival of novel agents compared to placebo according to the subgroup analysis evaluated in the meta-analysis. Table 1S: hazard ratio and confidence interval of overall survival for different subgroup analysis.
