# Supplementary material for: Defining the Ideal Patient with Hepatocellular Carcinoma for Second-Line Treatment
Source: J Oncol. 2020 Jun 19;2020:8024124. doi: 10.1155/2020/8024124 (PMC7321521; doi:10.1155/2020/8024124)
Supplement: Supplementary Materials — Figures of subgroup analysis for overall survival of novel agents compared to placebo according to the subgroup analysis evaluated in the meta-analysis. Table 1S: hazard ratio and confidence interval of overall survival for different subgroup analysis. [file 8024124.f1.docx]

Subgroup analysis for overall survival of novel agents compared to placebo according the subgroup analysis of:

1. **Age**


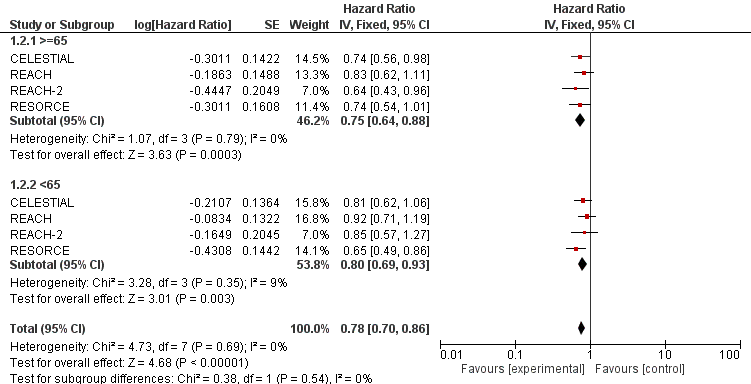


1. **Sex**


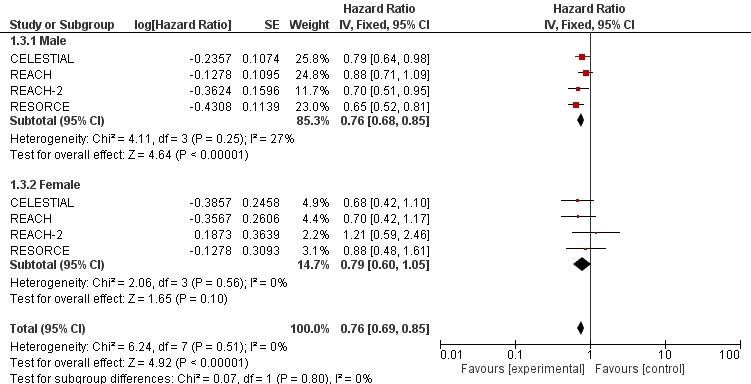


1. **ECOG**


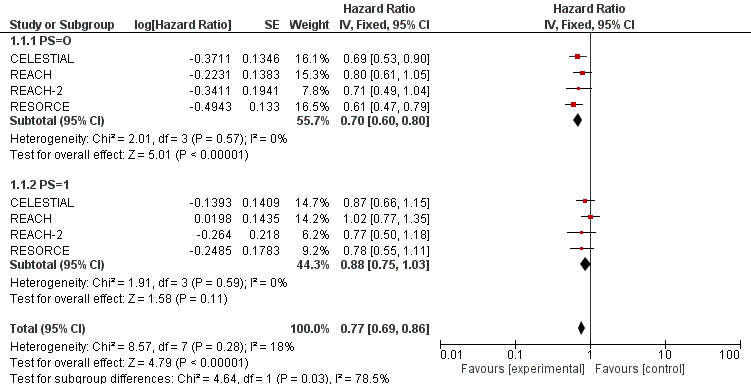


1. **Alpha-fetoprotein**


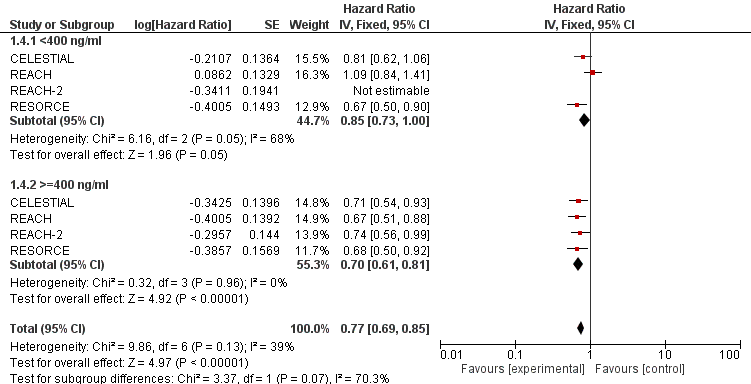


1. **Macrovascular invasion**


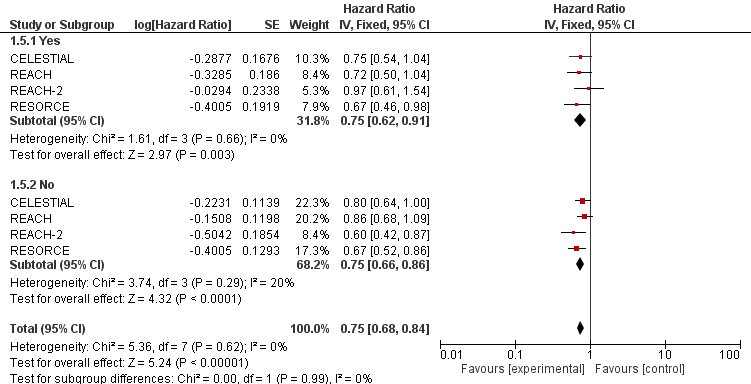


1. **Extra-hepatic metastases**


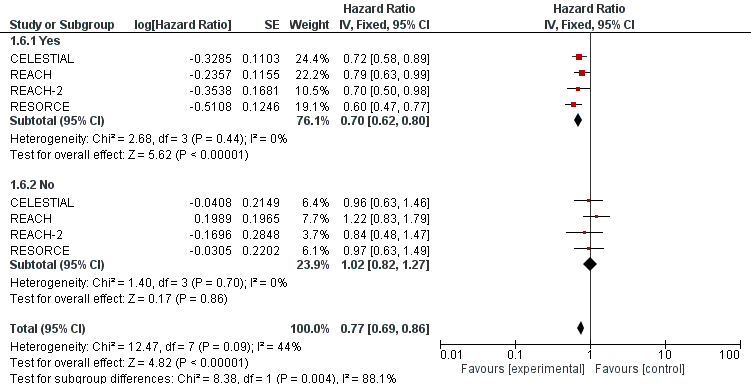


1. **Virus**


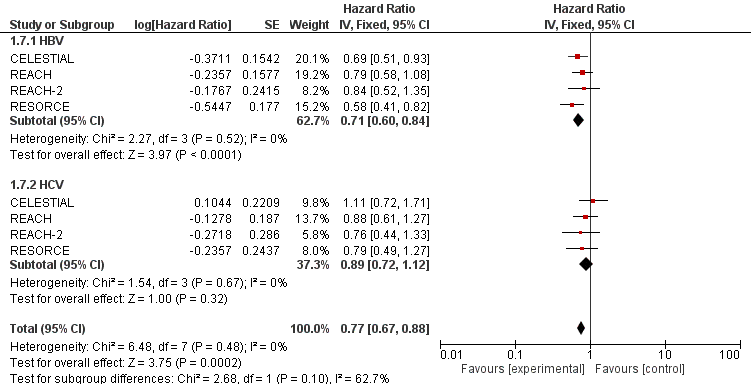


**Table 1S**. Hazard ratio and confidence interval of overall survival different subgroup analysis.

|  | **CELESTIAL** | **REACH** | **REACH-2** | **RESORCE** |
| --- | --- | --- | --- | --- |
| Age < 65 years  Age ≥ 65 years | 0.81 (0.62-1.05)  0.74 (0.56-0.97) | 0.92 (0.71-1.18)  0.83 (0.62-1.11) | 0.85 (0.57-1.27)  0.64 (0.43-0.96) | 0.65 (0.49-0.87)  0.74 (0.54-1.02) |
| Male  Female | 0.79 (0.64-0.97)  0.68 (0.42-1.09) | 0.88 (0.71-1.08)  0.70 (0.42-1.16) | 0.70 (0.51-0.95)  1.21 (0.59-2.46) | 0.65 (0.52-0.82)  0.88 (0.48-1.62) |
| PS ECOG: 0  PS ECOG: 1 | 0.69 (0.53-0.89)  0.87 (0.66-1.14) | 0.80 (0.61-1.04)  1.02 (0.77-1.35) | 0.71 (0.49-1.04)  0.77 (0.50-1.18) | 0.61 (0.47-0.80)  0.78 (0.55-1.11) |
| Alpha-fetoprotein  <400 ng/ml  ≥400 ng/ml | 0.81 (0.62-1.04)  0.71 (0.54-0.94) | 1.09 (0.84-1.43)  0.67 (0.51-0.90) | N.A  0.74 (0.56-0.99) | 0.67 (0.50-0.90)  0.68 (0.50-0.92) |
| Macrovascular invasion  Yes  No | 0.75 (0.54-1.03)  0.80 (0.64-1.01) | 0.72 (0.50-1.03)  0.86 (0.68-1.08) | 0.97 (0.61-1.53)  0.60 (0.42-0.87) | 0.67 (0.46-0.98)  0.67 (0.52-0.86) |
| Extra-hepatic metastases  Yes  No | 0.72 (0.58-0.89)  0.96 (0.63-1.46) | 0.79 (0.63-0.98)  1.22 (0.83-1.79) | 0.70 (0.50-0.98)  0.84 (0.48-1.47) | 0.60 (0.47-0.77)  0.97 (0.63-1.48) |
| HBV  HCV | 0.69 (0.51-0.94)  1.11 (0.72-1.71) | 0.79 (0.58-1.07)  0.88 (0.61-1.26) | 0.84 (0.52-1.35)  0.76 (0.43-1.33) | 0.58 (0.41-0.82)  0.79 (0.49-1.26) |
